# Supplementary material for: Complete and efficient conversion of plant cell wall hemicellulose into high-value bioproducts by engineered yeast
Source: Nat Commun. 2021 Aug 17;12:4975. doi: 10.1038/s41467-021-25241-y (PMC8371099; doi:10.1038/s41467-021-25241-y)
Supplement: Supplementary file 1 — Supplementary Information [file 41467_2021_25241_MOESM1_ESM.pdf]

Complete and efficient conversion of plant cell wall hemicellulose into high-value bioproducts by engineered yeast

Sun *et al.*

Supplementary Table 1. The list of strains used in this study.

Strains	Description	Source
E.coli DH5α	*F-, ϕ80d, lacZΔM15, endA1, recA1, hsdR17 (rK-mK), supE44, thi-1, gyrA96, relA1*	Invitrogen, CA
SR7	D452-2 integrated with multiple copies of *XYL1*, *XYL2*, and *XYL3*	[1]
SR8	Evolutionary engineered SR7 with the truncation of *ALD6* gene	[1]
SR8A	SR8 *ura3::URA3* YIplac211YB/I/E*, CS8:: pCCW12-coBlh-tCYC1	[2]
CT2-auxo	D452-2 *URA3*::*XYL123*, *PHO13*:: *XYL123*, *ALD6*:: *XYL123*, *leu2*, *his3*,*ura3*	[3]
CT2-4	CT2-auxo with *TRP1* disrupted	This study
CT2-con	CT2-4 *ura3*:: *URA3* pRS406, *leu2*:: *LEU2* pRS405, *trp1*:: *TRP1* pRS404, *his3*:: *HIS3* pRS403	This study
Tal1	CT2-4 *his3*:: *HIS3* pRS403-2PS	This study
Tal2	Tal1 *ura3*:: *URA3* pRS 406-2PS	This study
Tal3	Tal2 *leu2*:: *LEU2* pRS 405-2PS	This study
Tal4	Tal3 *trp1*:: *TRP1* pRS 404-2PS	This study

Supplementary Table 2. The list of plasmids used in this study.

Plasmids	Description	Source
Cas9-NAT	Cas9 expression plasmid, NAT1 marker	[4]
gRNA-trp-HYB	*TRP1* disruption gRNA cassette, HyB marker	[4]
pRS403	Integrative vector with HIS3 marker	[5]
pRS404	Integrative vector with TRP1 marker	[5]
pRS405	Integrative vector with LEU2 marker	[5]
pRS406	Integrative vector with URA3 marker	[5]
pRS426-pCCW12	pRS426 plasmid with CCW12 promoter and CYC1terminator	[2]
pRS426-2PS	pRS426-pCCW12 plasmid with *co-2PS* inserted between *Xho I* and *BamH I*	This study
pRS403-2PS	pRS403 plasmid carrying pCCW12-2PS-CYC1 cassette	This study
pRS404-2PS	pRS404 plasmid carrying pCCW12-2PS-CYC1 cassette	This study
pRS405-2PS	pRS405 plasmid carrying pCCW12-2PS-CYC1 cassette	This study
pRS406-2PS	pRS406 plasmid carrying pCCW12-2PS-CYC1 cassette	This study

Supplementary Table 3. The list of primers and gBlock used in this study.

Primers/gBlock	Description	Source
TRP1donor-U	TCCGATGCTGACTTGCTGGGTATTATATGTGTGTAAAATAGAAAGAG<br>AACAATTGACCCG	[4]
TRP1donor-D	TACAAGACTTGAAATTTTCCTTGCAATAACCGGGTCAATTGTTCTCTT<br>TCTATTTTACAC	[4]
TRP1-Seq-U	CATTGGTGACTATTGAGCAC	[5]
TRP1-Seq-D	CAAAAGGCCTGCAGGCAAGT	[5]
2PS-Amp-U	CGGGATCCAAAACAATGGGATCATATAGCAGCGA	[5]
2PS-Amp-D	CCGCTCGAGTTAGTTTCCATTTGCGACTGCCGCCG	[5]
Vector-U	ACGTCTCACGGATCGTATATGCCGTAGCGACAATCTAAGAACTATGC<br>GAGGACACGCTAGGTCATAGCTGTTTCC	[2]
Vector-D	GTTGAACATTCTTAGGCTGGTCGAATCATTTAGACACGGGCATCGTC<br>CTCTCGAAAGGTGACTGGCCGTCGTTTTAC	This study
Insert-U	CACCTTTCGAGAGGACGATGCCCGTGTCTAAATGATTCGACCAGCCT<br>AAGAATGTTCAACTAATACGACTCACTATAGG	This study
Insert-D	CTAGCGTGTCCTCGCATAGTTCTTAGATTGTCGCTACGGCATATACG<br>ATCCGTGAGACGTAATTAACCCTCACTAAAGG	This study
gBlock-co2PS	ATGGGATCATATAGCAGCGATGACGTAGAAGTAATTAGAGAAGCAG<br>GAAGGGCGCAGGGTTTAGCTACGATACTAGCCATAGGAACCGCCAC<br>CCCTCCCAATTGCGTAGCGCAAGCCGATTATGCAGACTATTATTTC<br>AGGGTTACTAAAAGTGAGCACATGGTTGATCTTAAGGAGAAATTTA<br>AGCGTATCTGCGAAAAGACGGCCATCAAAAAGAGGTATTTGGCGCT<br>AACCGAGGACTACTTACAAGAAAATCCGACGATGTGTGAGTTCATG<br>GCTCCTTCTTTGAATGCGAGGCAAGATCTTGTAGTCACGGGTGTCC<br>CTATGTTAGGGAAAGAGGCTGCAGTTAAGGCCATCGACGAGTGGGG<br>GCTGCCGAAATCCAAAATTACTCATCTTATTTTCTGCACAACGGCG<br>GGAGTTGATATGCCTGGGGCCGACTATCAACTGGTGAAACTGCTGG<br>GCCTAAGTCCGAGTGTCAAGAGGTATATGCTATACCAACAAGGGTG<br>CGCGGCAGGCGGTACGGTCTTAAGACTGGCAAAAGACTTAGCTGAA<br>AACAATAAGGGATCACGTGTCTTGATCGTATGTTCTGAAATCACGG<br>CAATCCTATTCCATGGCCCCAACGAAAATCATCTAGACTCATTGGT<br>TGCACAAGCTCTGTTTGGCGACGGCGCCGCCGCTTTGATAGTCGGA<br>TCTGGACCACACTTAGCTGTGGAAAGGCCAATCTTTGAAATTGTCT<br>CCACGGACCAAACTATCCTGCCGGATACGGAAAAGGCGATGAAGCT<br>ACATCTTAGAGAAGGGGGGCTAACGTTCCAGTTACACCGTGACGTT<br>CCCCTAATGGTTGCTAAGAATATAGAAAATGCCGCCGAGAAAGCTC<br>TGTCCCCGCTAGGCATCACTGATTGGAACTCCGTGTTTTGGATGGT<br>TCATCCCGGGGGCAGGGCCATATTAGATCAAGTCGAGCGTAAATTG<br>AATCTAAAGGAGGACAAGTTGAGAGCATCCAGGCACGTCCTTAGTG<br>AGTACGGGAATCTAATTTCTGCCTGCGTCTTGTTTATAATAGATGA<br>GGTTCGTAAAAGATCAATGGCGGAGGGAAAAAGTACCACTGGTGAG<br>GGTCTTGATTGTGGAGTGCTTTTTGGTTTTGGGCCAGGTATGACCG<br>TAGAAACGGTGGTTCTGCGTAGTGTACGTGTTACGGCGGCAGTCGC<br>AAATGGAAACTAA	This study

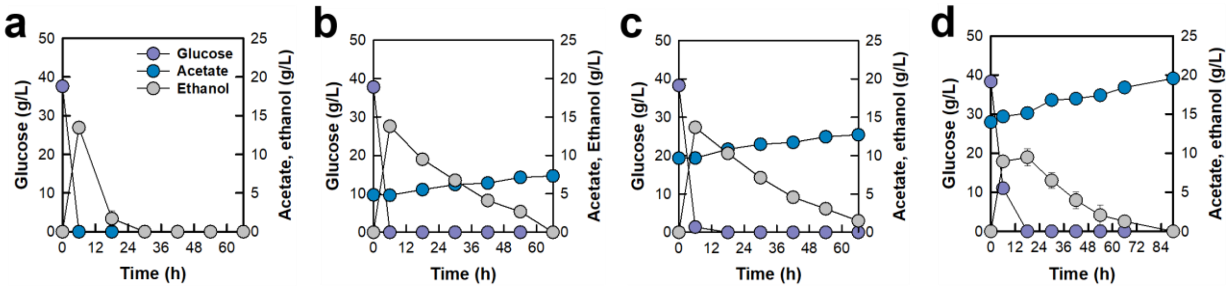

Supplementary Figure 1. Ethanol reassimilation by SR7 strain with wildtype *ALD6* gene in glucose cultures with acetate supplementation of 0 g/L (**a**), 5 g/L (**b**), 10 g/L (**c**), and 15 g/L (**d**). Data are presented as mean value and standard deviations of three independent biological replicates. Source data are provided as a Source Data file.

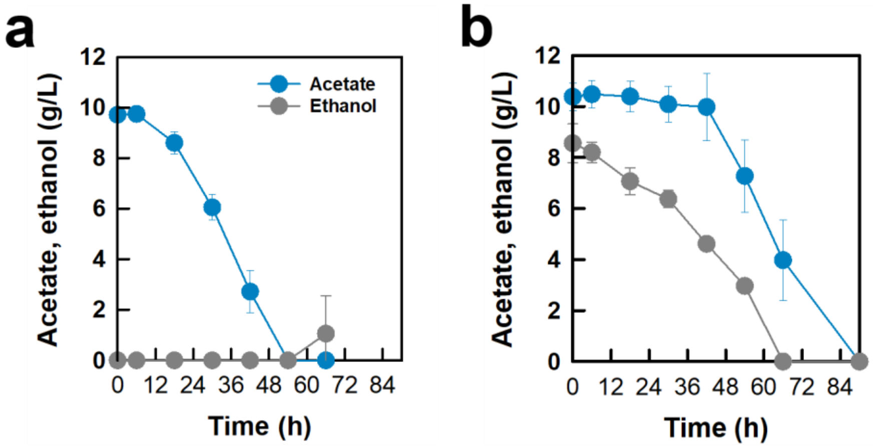

Supplementary Figure 2. Ethanol repression on aerobic assimilation of acetate. **a** Culture profile of SR8 strain on 10 g/L acetate. **b** Culture profile of SR8 strain on 10 g/L acetate and 9 g/L ethanol. Data are presented as mean value and standard deviations of three independent biological replicates. Source data are provided as a Source Data file.

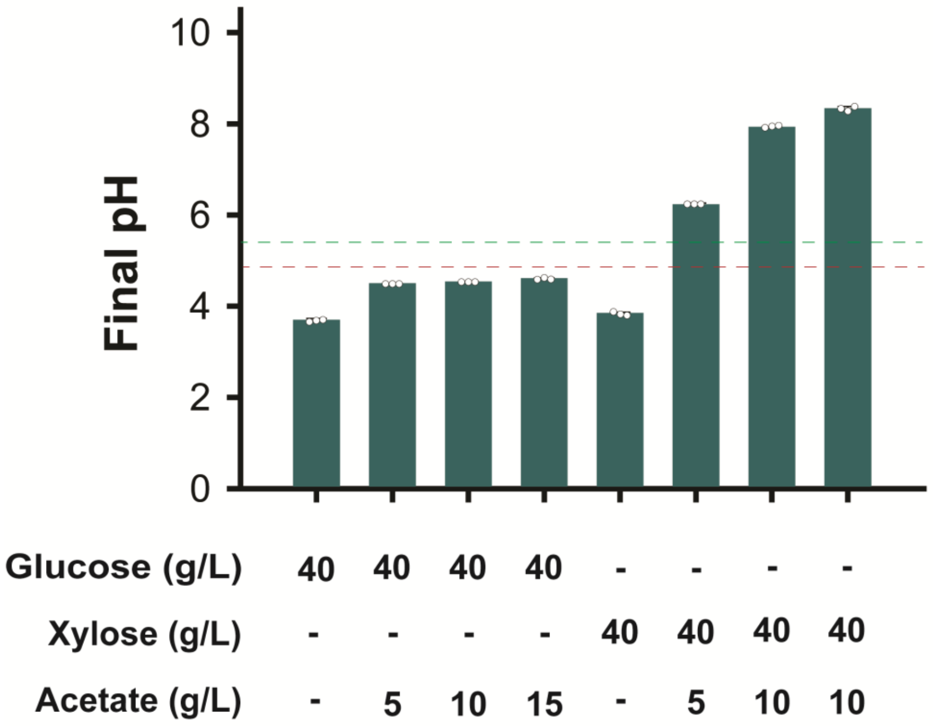

Supplementary Figure 3. Increased pH in culture broths with the consumption of acetate. The bar height represents final pH of culture broths with different substrates. The dash line in green represents initial pH of 5.5. The dash in red represents the pKa of acetic acid (4.76). Data are presented as mean value and standard deviations of three independent biological replicates. Source data are provided as a Source Data file.

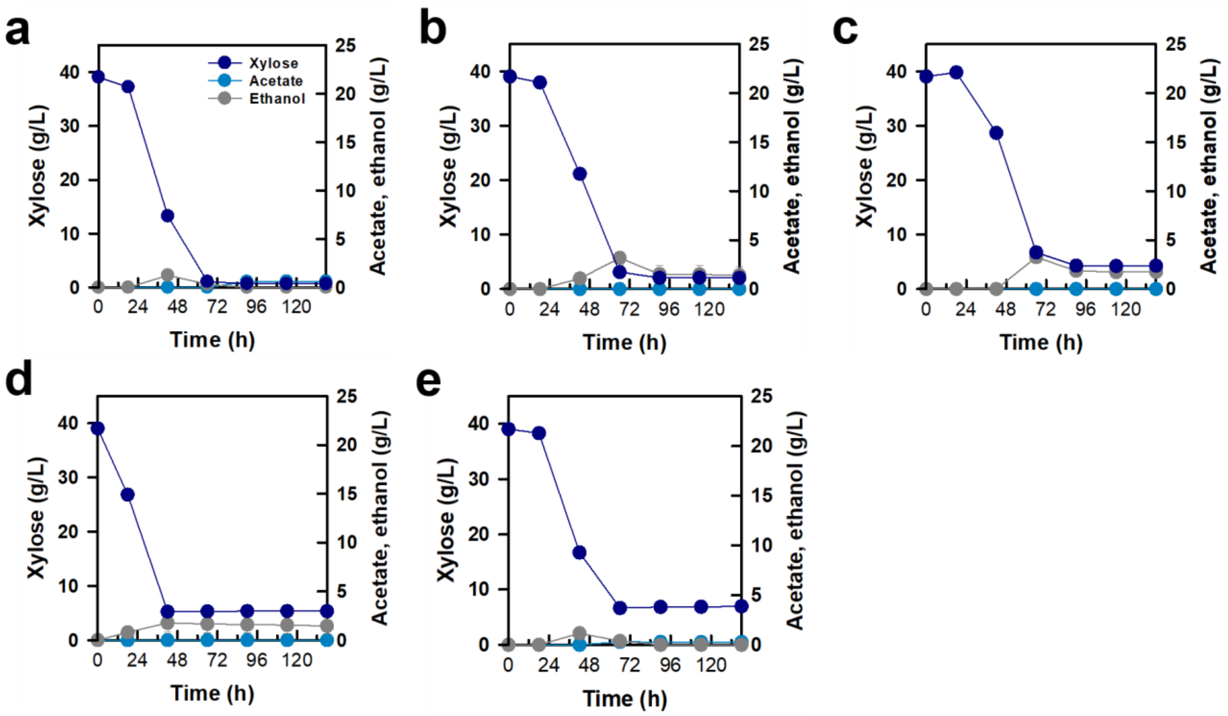

Supplementary Figure 4. The effect of increased copies of *2PS* gene on xylose consumption. The figures present fermentation profiles of CT2-Con (**a**), Tal1 (**b**), Tal2 (**c**), Tal3 (**d**) and Tal4 (**e**) on xylose. Data are presented as mean value and standard deviations of three independent biological replicates. Source data are provided as a Source Data file.

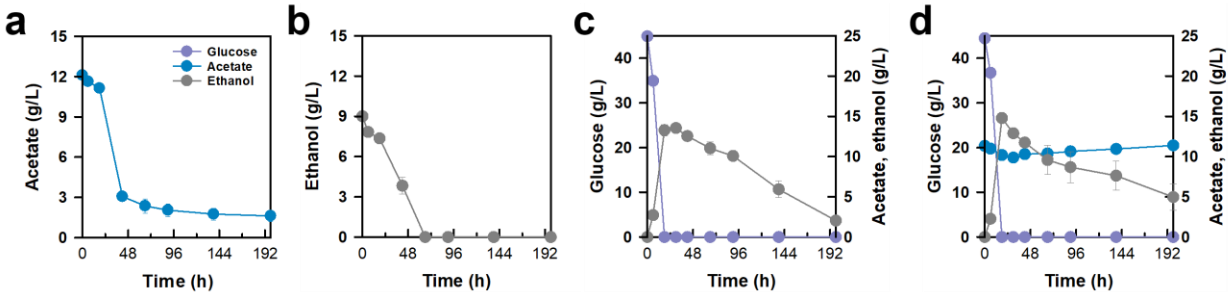

Supplementary Figure 5. Fermentation profiles of Tal4 on 12 g/L acetate (**a**), 9 g/L ethanol (**b**), 45 g/L glucose (**c**) and 45 g/L glucose with 11 g/L acetate (**d**). Data are presented as mean value and standard deviations of three independent biological replicates. Source data are provided as a Source Data file.

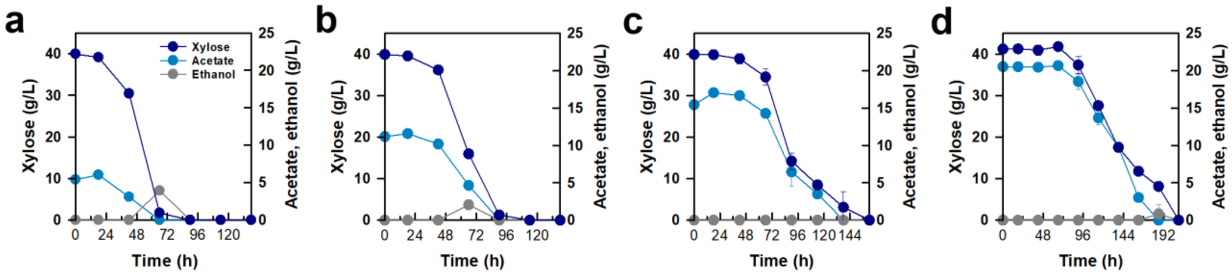

Supplementary Figure 6. Co-utilization of xylose and acetate by Tal4 strain. The figures present the fermentation profiles of Tal4 on 40 g/L xylose with supplementation of 5 g/L (**a**), 10 g/L (**b**), 15 g/L (**c**) and 20 g/L (**d**) acetate. Data are presented as mean value and standard deviations of three independent biological replicates. Source data are provided as a Source Data file.

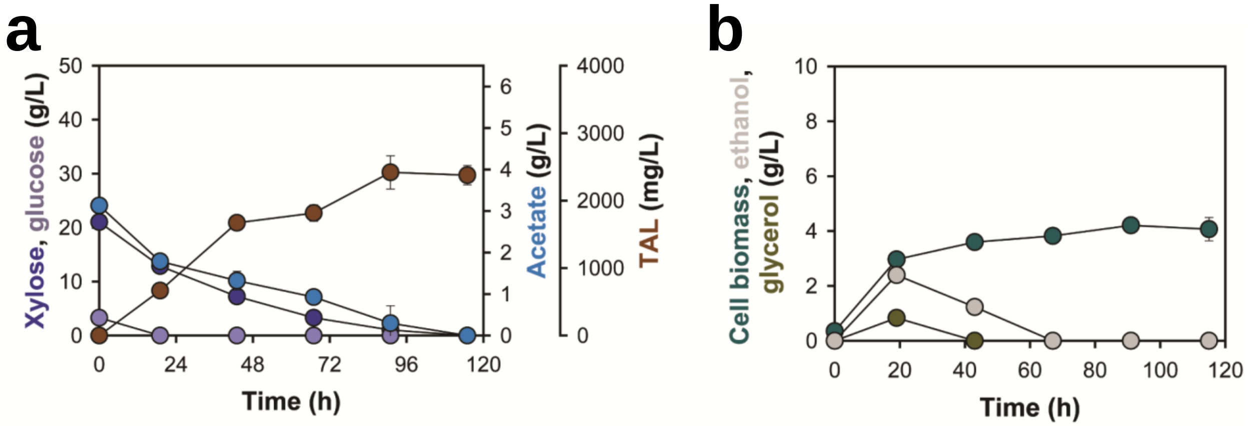

Supplementary Figure 7. Production of TAL by the Tal4 strain in unconcentrated switchgrass hemicellulose hydrolysate. **a** Profiles of TAL, xylose, acetate, and glucose concentrations. **b** Profiles of cell biomass, ethanol, and glycerol concentrations. Data are presented as mean value and standard deviations of three independent biological replicates. Source data are provided as a Source Data file.

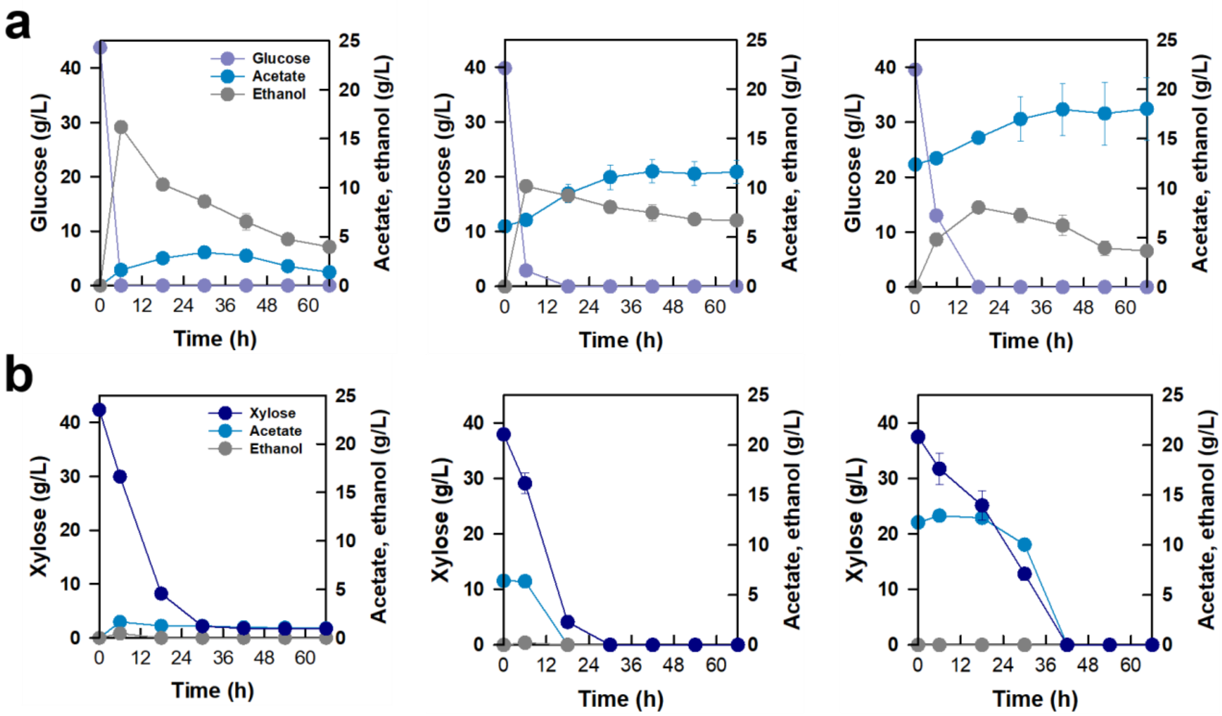

Supplementary Figure 8. Co-utilization of xylose and acetate by SR8A strain. **a** Glucose cultures of SR8A strain with acetate supplementation of 0, 6 and 12 g/L. **b** Xylose cultures of SR8A strain with acetate supplementation of 0, 6 and 12 g/L (**f**). Data are presented as mean value and standard deviations of three independent biological replicates. Source data are provided as a Source Data file.

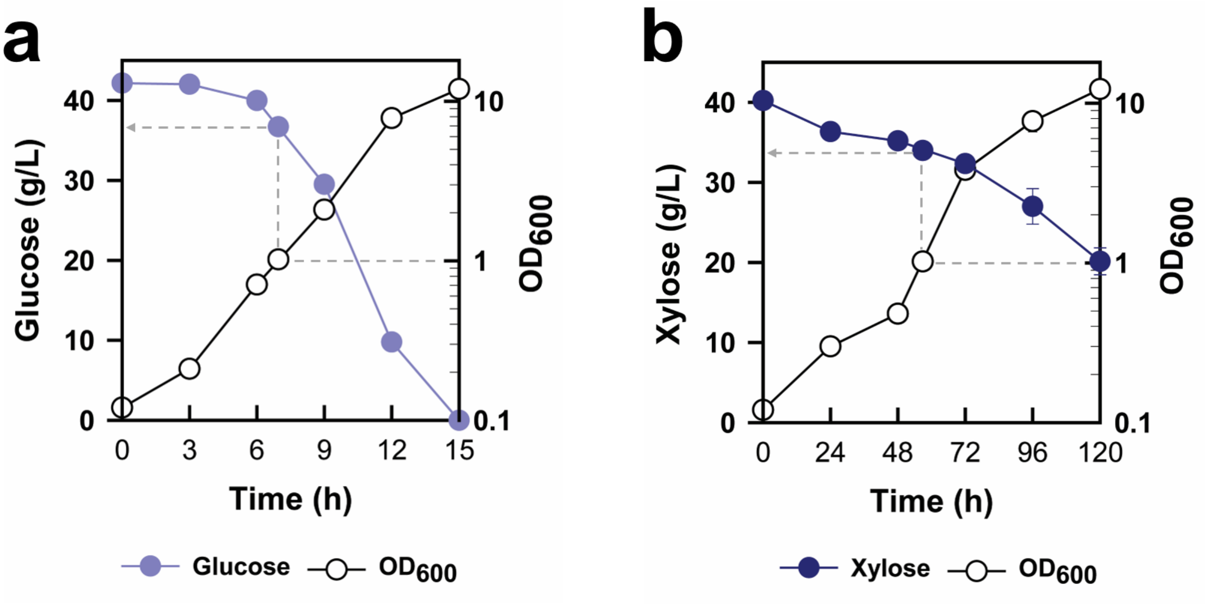

Supplementary Figure 9. Culturing profiles of the SR7 strain in 40 g/L glucose (**a**) and 40 g/L xylose (**b**) for RNA sequencing. The dash lines point out OD_{600nm} and sugar concentrations by the time of cell sampling for RNA extraction. Data are presented as mean value and standard deviations of three independent biological replicates. Source data are provided as a Source Data file.

Supplementary references

1. Kim, S. R. *et al.* Rational and evolutionary engineering approaches uncover a small set of genetic changes efficient for rapid xylose fermentation in *Saccharomyces cerevisiae*. *PLoS One* **8**, e57048 (2013).
2. Sun, L., Kwak, S. & Jin, Y.-S. Vitamin A production by engineered *Saccharomyces cerevisiae* from xylose *via* two-phase *in situ* extraction. *ACS Synth. Biol.* **8**, 2131–2140 (2019).
3. Tsai, C.-S. *et al.* Rapid and marker-free refactoring of xylose-fermenting yeast strains with Cas9/CRISPR. *Biotechnol. Bioeng.* **112**, 2406–2411 (2015).
4. Zhang, G.-C. *et al.* Construction of a quadruple auxotrophic mutant of an industrial polyploid *Saccharomyces cerevisiae* strain by using RNA-guided Cas9 nuclease. *Appl. Environ. Microbiol.* **80**, 7694–7701 (2014).
5. Sikorski, R. S. & Hieter, P. A system of shuttle vectors and yeast host strains designed for efficient manipulation of DNA in *Saccharomyces cerevisiae*. *Genetics* **122**, 19–27 (1989).
